# The Scalp Confounds Near-Infrared Signal from Rat Brain Following Innocuous and Noxious Stimulation

**DOI:** 10.3390/brainsci5040387

**Published:** 2015-09-29

**Authors:** Ji-Wei He, Hanli Liu, Yuan Bo Peng

**Affiliations:** 1 Departments of Psychology, University of Texas at Arlington, Arlington, TX 76019, USA; E-Mail: ypeng@uta.edu; 2 Department of Neurological Surgery, University of California San Francisco, 1700 Owens Street, San Francisco, CA 94158, USA; 3 Department of Bioengineering, University of Texas at Arlington, Arlington, TX 76019, USA; E-Mail: hanli@uta.edu

**Keywords:** pain, oxygenated hemoglobin, deoxygenated hemoglobin, total blood volume change, oxygenation, neurovascular coupling, sympathetic, autonomic arousal

## Abstract

Functional near-infrared imaging (fNIRI) is a non-invasive, low-cost and highly portable technique for assessing brain activity and functions. Both clinical and experimental evidence suggest that fNIRI is able to assess brain activity at associated regions during pain processing, indicating a strong possibility of using fNIRI-derived brain activity pattern as a biomarker for pain. However, it remains unclear how, especially in small animals, the scalp influences fNIRI signal in pain processing. Previously, we have shown that the use of a multi-channel system improves the spatial resolution of fNIRI in rats (without the scalp) during pain processing. Our current work is to investigate a scalp effect by comparing with new data from rats with the scalp during innocuous or noxious stimulation (*n* = 6). Results showed remarkable stimulus-dependent differences between the no-scalp and intact-scalp groups. In conclusion, the scalp confounded the fNIRI signal in pain processing likely via an autonomic mechanism; the scalp effect should be a critical factor in image reconstruction and data interpretation.

## 1. Introduction

Since its birth in the 1970s, functional near-infrared imaging (fNIRI) has been increasingly utilized to measure brain activity in healthy and pathological conditions [1,2,3,4,5] (for reviews see [6,7]). Compared to other non-invasive imaging technologies, e.g., functional magnetic resonance imaging (fMRI), positron emission tomography (PET) and magnetoencephalography (MEG), fNIRI is much more portable yet economically efficient, and is, thus, considered as a potential bedside physiological assessment of brain functions in clinic.

Although the current fNIRI techniques have been greatly improved in many ways, the poor spatial resolution remains a caveat that limits fNIRI application for research and clinical purposes. To improve the spatial resolution of fNIRI, multi-channel systems have been developed [8,9,10] with a rationale that has led to tremendous successes in the early developments of MRI and PET. However, due to the heterogeneous effects of the brain, scalp, and skull on light absorption and scattering, the reconstruction of the fNIRI-derived brain activity is mathematically challenging [11]. Nevertheless, as an early success we have shown that, in rats (without the scalp), a multi-channel system is able to provide a whole-brain assessment of brain activity during somatosensory or pain processing with a better spatial resolution compared to the conventional single channel-based topography [12].

In our previous study [12], one critical question remained unanswered: whether the scalp influences fNIRI signal in pain processing and the subsequent reconstruction. Unlike tactile sensation, visual perception or motor activity, pain (induced by noxious stimulus) is often associated with an autonomic arousal as characterized by an increase in heart rate, paleness, pupil dilation, and an increase in blood pressure [13,14,15]. As such the autonomic arousal is systemic and is believed to be, in part, the underlying mechanism of the fight-or-flight response in a life-or-death situation [13]. Therefore, the effect of the scalp may be more profound upon an autonomic arousal (e.g., when pain is perceived) than a non-arousal case. Indeed, early studies raised a concern about the effect of the scalp on fNIRI signal [16,17]; a recent work in humans suggested that, in pain processing, the scalp can confound fNIRI signals [18].

The objective of our current work is to investigate how the scalp influences fNIRI signal in pain processing using a multi-channel system and a tomographic algorithm for image reconstruction. Our results showed a clear scalp-effect on fNIRI signal in a stimulus-dependent manner. In particular, between the no-scalp [12] and intact-scalp groups, brief stimuli (e.g., brushing or pinching) produced no similarity in regional hemodynamic response; whereas the formalin injection produced some similar responses only in Phase I (first 10 min). These results strongly support the notion that the scalp can confound fNIRI-derived cerebrovascular response in pain processing.

## 2. Materials and Methods

### 2.1. Instrumentation

**Figure 1:**
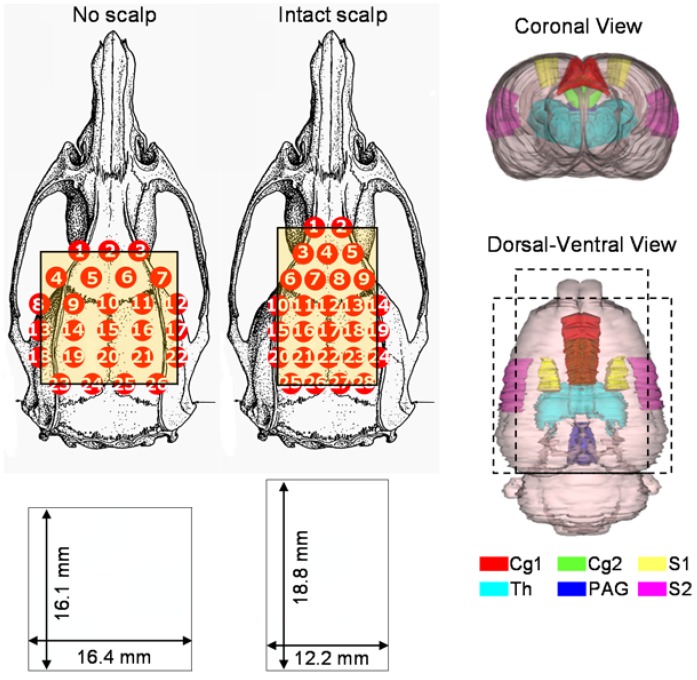
Optode-array placements in the no-scalp (**left**; [12]) and intact-scalp (**right**) groups. The sizes (**bottom**) of reconstructed images were dependent on array placements. To better illustrate functional areas in reconstructed images, six associated brain regions are segmented in a 3D rat atlas (Atlas3D, NeSys, Oslo, Norway), namely the dorsal/ventral anterior cingulate cortex (Cg1/2), primary somatosensory cortex for hindlimb (S1), secondary somatosensory cortex (S2), thalamus (Th), and periaqueductal gray (PAG).

A commercialized continuous-wave (CW) near-infrared imager, Dynamic Near-Infrared Optical Tomography (DYNOT, NIRx Medical Technologies, New York, USA), was used to measure hemodynamic changes of the rat brain. Twenty-eight optodes (2 mm in diameter; each is bifurcated with two fiber bundles, one to the light source and the other to the detector) were held in place by a plastic frame, and were positioned on the top of a shaved rat head, where the posterior row of the optode array was aligned with the external orifices of the ears. The optode placements are shown in Figure 1 for the no-scalp (right; [12]) and intact-scalp (left) groups, respectively. Optical signals at two wavelengths (760 and 830 nm) were acquired simultaneously and independently. As each optode can work as a light source or a detector, there were 28 × 27 combinations of source-detector channels in data acquisition. Detailed procedures of data acquisition and image reconstruction can be found elsewhere [12,19,20]. A brief description is provided as follows. Data points from each source-detector channel were normalized by corresponding data points from a source-detector channel where the source and detector were co-located. Using HOMER [21], 756 (28 × 27) source-detector channels (excluding source-detector co-located channels) were imported; the cutoff for low-pass filter was set at 0.1 Hz; and the baseline period was defined as 0–5 s prior to stimulation onset. For mechanical stimuli, five consecutive trials were averaged and used for reconstruction with the L2-norm regularization [22]. Finally, after image reconstruction (by HOMER), light-intensity-based images at 2 wavelengths were converted to hemodynamic changes [23] at each pixel using Matlab (MathWorks, USA). Results of our phantom experiments using either 28- or 26-optode-array indicated that both arrays reliably detected the location of a phantom absorber.

It is noteworthy that the baseline period (0–5 s prior to the stimulation onset) was chosen to be the same as our previous study [12] in the hopes of making a simple comparison for speculating a scalp effect. However, it would be interesting to compare hemodynamic responses between other baseline periods (e.g., 5 min) and the post-injection period up to 1 h. Nonetheless, it is recently known that the default mode network at a resting state in both rats and humans is very active, giving rise to detectable hemodynamic signals. Thus, a 5-min baseline may not be an optimal temporal window for comparison to assess a stimulus-induced response.

### 2.2. Animal Preparations

Six male Sprague-Dawley rats were used with a mean age of 71.5 ± 2.9 days (mean ± SEM) and a mean weight of 322.8 ± 9.8 g. All animals were anesthetized by intraperitoneal injection of pentobarbital sodium solution (50 mg/kg) and additional injections were made every hour to maintain anesthesia. All procedures were approved by the Institutional Animal Care and Use Committee (IACUC) at the University of Texas at Arlington. Procedures also followed the guidelines described by the Committee for Research and Ethical Issues of International Association for the Study of Pain [24].

### 2.3. Brief Mechanical Stimulations

Innocuous brushing and noxious pinching were applied to the plantar surface of rat hindpaw unilaterally with five trials. Brushing was referred as to briefly sweeping a camel-hair bush in a rhythmic fashion for 10 s. Pinching was referred as to constantly clamping the hindpaw with a straight arterial bulldog clamp (3 cm in length) for 10 s. Five consecutive trials of brushing or pinching were applied to the same side of the paw with an interval of 2 min. The side of stimulation was selected pseudorandomly. Three rats received the stimuli on the left hindpaw, and the other 3 on the right. Pinching was applied 5 min after all brushing trials were complete to the same paw for each animal. Data from 5 blocks were then superimposed with respect to stimulation onset prior to image reconstruction.

### 2.4. A Long-Lasting Noxious Stimulation by Formalin Injection

After mechanical stimulation and a 5 min baseline measurement, formalin solution (50 μL, 3%) was injected subcutaneously into the center of the plantar area in the other hindpaw not receiving mechanical stimulation. A 1-h continuous recording was obtained after injection. One rat was excluded due to body movement right after injection (possibly causing optode displacements).

### 2.5. Statistical Analysis

Directional one-sample *t*-tests were used for each of 21-by-21 pixels from a series of hemodynamic images to test relative changes in regional oxy- (HbO), deoxy- (Hb), and total-hemoglobin concentration (HbT) at some selected time points. Pixel-based *t*-tests were performed in Matlab (MathWorks, US). Directional one-sample *t*-tests were also used to test relative changes in HbO, Hb and HbT at regions of interest (ROIs) in SPSS (SPSS Statistics 17.0, SPSS, Chicago, USA). Three ROIs for the new intact-scalp group were defined by regional maximum/minimum of oxy-hemoglobin values in order to highlight the dominant responses, including a middle posterior area with an increase in HbO at 1 min after formalin injection (also showing a decrease in HbO during brushing), a central ipsilateral area with a decrease in Hb at 1 min after formalin injection, and finally a contralateral area with a decrease in HbO during pinching or brushing. To assess a scalp-effect on regional hemodynamic changes, correspondingly adjacent ROIs in the no-scalp group [12] were used. As brushing failed to induce a statistically significant change in lateral areas [12], we used the same ROI during pinching to demonstrate a region-specific hemodynamic difference. The alpha was set at 0.05. To calculate statistical power of directional one-sample *t-test*, we used a command *power.t.test* with a one-side assumption in R 3.2.2 for all significant differences [25]. Error bars in the time series indicate SEM.

## 3. Results

Six rats were used in the intact-scalp group. However, one (the first) rat which received only one brushing trial was excluded for analysis of a brushing effect (sample size: *n* = 5), and one rat which slightly twitched the head shortly after the formalin injection was excluded for analysis of a formalin effect (sample size: *n* = 5), whereas for analysis of a pinching effect all rats were included (sample size: *n* = 6). To better visualize the effect of the scalp on fNIRI signal, hemodynamic images and time series of regional hemodynamic changes at several representative ROIs are organized in a parallel manner from the no-scalp [12] and intact-scalp groups. The reuse of our own data (permission granted) from the no-scalp group [12] is necessary to identify and characterize a scalp effect.

### 3.1. Formalin-Induced Hemodynamic Changes

**Figure 2:**
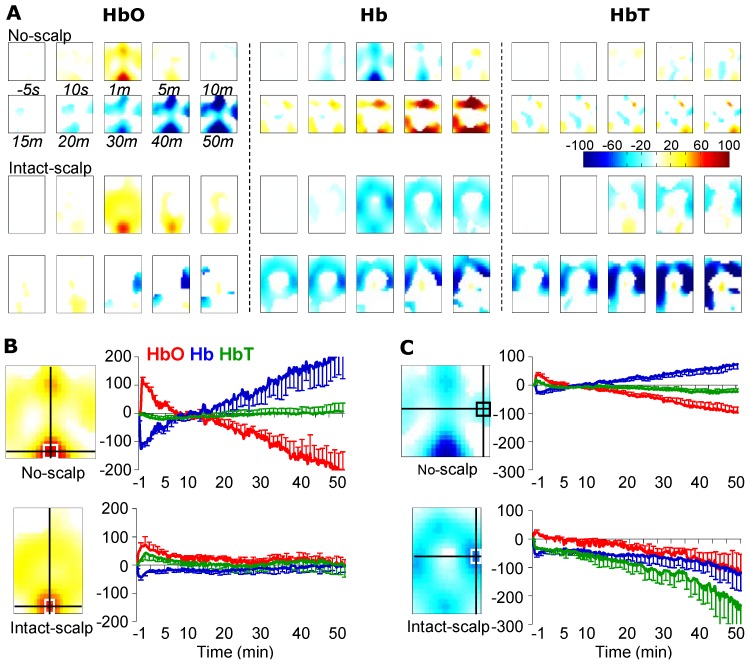
Formalin-induced hemodynamic changes in the no-scalp [12] and intact-scalp groups. (**A**) reconstructed images of hemodynamic changes at typical time points. The intensity of change (microMolar) is color-coded; only statistically significant changes (per pixel) are shown in color; (**B**) time series of hemodynamic changes at a middle posterior ROI (top: no-scalp [12]; bottom: intact-scalp). Locations of ROIs were determined in example images (left insets) at 1 min post injection in HbO; (**C**) time series of hemodynamic changes at a center ipsilateral ROI. Locations of ROIs were determined in example images (left insets) at 1 min post injection in Hb. In each image, top: rostral; bottom: caudal; right: ipsilateral; left: contralateral. Error bar: SEM.

Figure 2A illustrates reconstructed hemodynamic changes from the no-scalp and intact-scalp groups at typical time points post injection. In both groups, there appeared, likewise, an increase in HbO and a concomitant decrease in Hb immediately after the injection (in Phase I; first 10 min) at similar locations. As shown in Figure 2B-C, averages of the first 5 min at two ROIs indicated the canonical hemodynamic pattern (*i.e.*, HbO ↑ and Hb ↓) from both groups. For instance, at the middle posterior ROI, HbO were increased (no-scalp: *t*(5) = 6.69, *p* < 0.001, *power* = 1; intact-scalp: *t*(4) = 3.07, *p* = 0.02, *power* = 0.87) whereas Hb were decreased (no-scalp: *t*(5) = −7.59, *p* < 0.001, *power* = 1; intact-scalp: *t*(4) = −4.95, *p* = 0.004, *power* = 1). However, in Phase II (15–60 min) there appeared hardly any similarities between the no-scalp and intact-scalp groups. For example, at the same middle posterior, ROI (Figure 2B), both HbO and Hb returned to baseline (averages of the last 10 min; HbO: *t*(4) = 0.59, *p* > 0.05; Hb: *t*(4) = −0.19, *p* > 0.05; HbT: *t*(4) = 0.23, *p* > 0.05). At a center ipsilateral ROI (Figure 2C), there appeared sustained decreases in HbO and Hb, causing a decrease in HbT at the end of recording (average of the last 10 min; *t*(4) = −2.18, *p* = 0.047, *power* = 0.64).

### 3.2. Pinching-Induced Hemodynamic Changes

**Figure 3:**
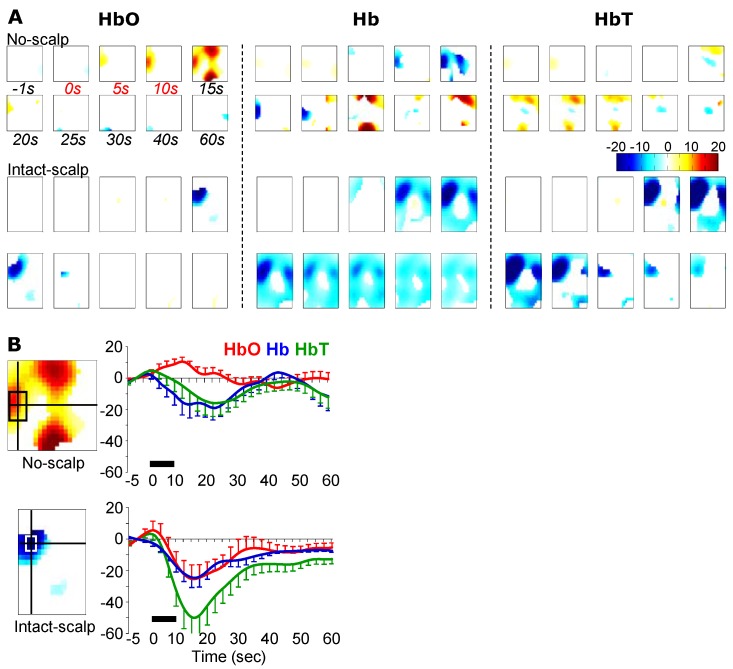
Pinching-induced hemodynamic changes in the no-scalp [12] and intact-scalp groups. (**A**) reconstructed images at typical time points. Unit: microMolar; (**B**) time series of hemodynamic changes at a center contralateral ROI. Locations of ROIs were determined in example images (left insets) at 15 s in HbO. Black bars: 10-s stimulation window.

Unlike the no-scalp group, the intact-scalp group did not show the canonical hemodynamic pattern anywhere (Figure 3A). Instead, decreases in HbO and Hb at a center contralateral ROI occurred (Figure 3B; averages of the first 30 s: HbO: *t*(5) = −2.26, *p* = 0.04, *power* = 0.69; Hb: *t*(5) = −4.30, *p* = 0.004, *power* = 0.99). Additionally, a strong decrease in Hb appeared to occur and progressively spread to almost the entire reconstructed image (Figure 3A).

### 3.3. Brushing-Induced Hemodynamic Changes

**Figure 4:**
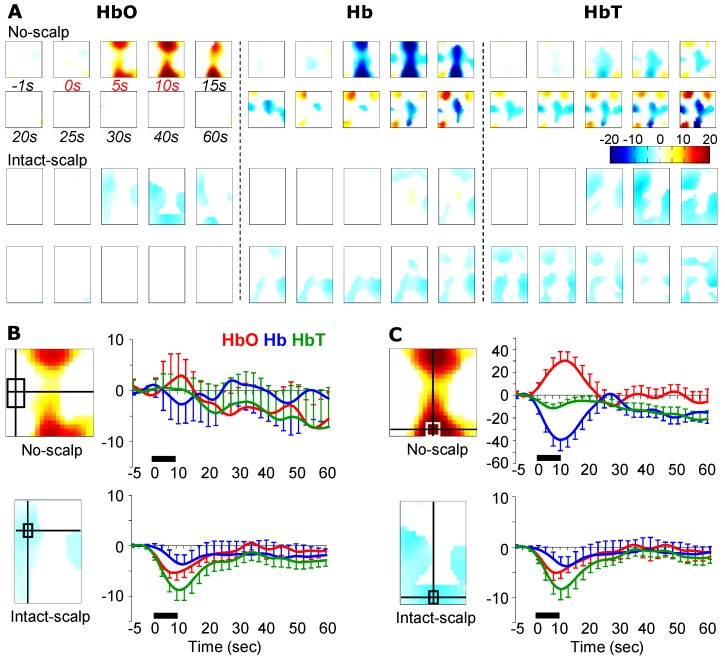
Brushing-induced hemodynamic changes in the no-scalp [12] and intact-scalp groups. (**A**) reconstructed images at typical time points. Unit: microMolar; (**B**) time series of hemodynamic changes at a contralateral ROI. Location of ROI in the intact-scalp group was determined in the example image (left inset) at 5 s in HbO; location of ROI in the no-scalp group is the same as in Figure 3B; (**C**) time series of hemodynamic changes at a middle posterior ROI. Locations of ROIs were determined in example images (left insets) at 10 s in HbO. Black bars: 10-s stimulation window.

Unlike the no-scalp group, the intact-scalp group did not show the canonical hemodynamic pattern anywhere (Figure 4A). But brushing indeed caused some hemodynamic changes at similar regions showing pinching-induced changes, albeit with smaller intensities (Figure 4B). For example, decreases in HbO and HbT were found in a contralateral ROI (ROI1; Figure 4B) as well as in a posterior ROI (ROI2; Figure 4C) from the intact-scalp group (averages of the first 30 s, HbO at ROI1: *t*(4) = −2.96, *p* = 0.02, *power* = 0.85; HbO at ROI2: *t*(4) = −2.18, *p* = 0.047, *power* = 0.64; HbT at ROI1: *t*(4)= −2.95, *p* = 0.02, *power* = 0.85; HbT at ROI2: *t*(4) = −3.00, *p* = 0.02, *power* = 0.86).

## 4. Discussion

We found clear differences between the no-scalp and intact-scalp groups in regional hemodynamic changes during somatosensory and pain processing, strongly supporting the notion that the scalp can influence fNIRI signal. More importantly, our results helped to address a critical concern about the validity of using tomographic algorithm for multi-channel-based image reconstruction. Although using tomographic algorithm improves the spatial resolution of fNIRI [12], the same tomographic algorithm failed to yield meaningful assessments of cerebrovascular response (except in the initial period after the formalin injection) when the scalp was intact. In brief, the scalp can confound fNIRI signal in a stimulus-dependent manner, and the scalp effect should be taken into careful consideration in image reconstruction and data interpretation.

### 4.1. Limitations

Our results may not be necessarily comparable to human data, for which there are at least three reasons.

First, we only used one tomographic algorithm (*i.e.*, HOMER) for image reconstruction in hope to make a fair comparison with our previous data (from the no-scalp group [12]). Possibly, our current results were method-dependent, and using another tomographic algorithm (with better modeling of the rat scalp) may create reconstructed images that are more similar to our previous data [12].

Second, our rodent setup differs from human setups. Particularly, the shortest source-detector distance of our setup was only ~3 mm, whereas in humans the distance usually ranges between 1 and 10 cm [10,26,27]. It is known that the precision of fNIRI signal in humans critically depends on the source-detector distance [10,18]; however, in rodents, the optimal source-detector distance for light to reach the rat brain without scalp contamination remains to be determined. In principle, as the combined thickness of rat skull and scalp is ~2 mm [28], the 3-mm short-separation channels should detect a large portion of hemodynamic signals coming from the scalp in the intact-scalp rat group. This contribution from extra-cranial layers is relatively strong due to a larger SNR compared to those signals from larger source-detector separation channels, the latter of which result from a true cerebrovascular activity. The confounding factor from the scalp can be reduced or minimized by regressing out the short-separation signals from the large-separation signals, as recently demonstrated in [18]. Our future studies may follow the same approach in order to obtain actual cerebral-hemodynamic activity in response to pain without the scalp contamination.

Third, due to the action of anesthetics, it is known that animals under general anesthesia may show a depressed neural activity in response to stimulation or in a resting state [29,30]. Thus, it remains unclear to what degree our data in anesthetized rats resemble the scalp effect on fNIRI signal in awake humans. In short, it is difficult or perhaps inappropriate to infer our experimental data in rats to the entire human fNIRI repertoire.

### 4.2. A Potential Mechanism: Autonomic Arousal

When the scalp was intact, it appeared that a decrease in HbT from a large, despite irregular, area was the dominant response pattern to all three stimuli, namely innocuous brushing, noxious pinching, and noxious formalin injection (Figure 2, Figure 3 and Figure 4). Additionally, the extent and duration of the decreases in HbT tended to positively correlate to stimulus intensities. As measured by the HbT change from the ROI of the global maximum, formalin injection (−250 microMolar; Figure 2C) > pinching (−50 microMolar; Figure 3B) > brushing (−10 microMolar; Figure 4B–C). As measured by the duration of HbT change, formalin injection (>50 min; Figure 2C) > pinching (>60 s; Figure 3B) > brushing (30 s; Figure 4B–C). Collectively, this stimulus-dependent pattern of HbT decrease and clear recovery periods following brief stimulations did not appear to be stimulation artifacts, but rather to manifest the physiological response to peripheral stimulation.

A possible explanation of the physiological response was an autonomic arousal, also speculated elsewhere [18]. The autonomic arousal mediated by the sympathetic and parasympathetic nervous systems (characterized by changes in heart rate, respiration rate, skin temperature, skin conductivity, *etc.*) often accompanies with emotional arousal [13]. During pain perception, negative emotion (e.g., unpleasantness, fear, depression, or anger) arises as a warning signal to the self, as well as others, to avoid the pain-causing incident. Similar to humans, animals also show pain-induced (or noxious stimulation-induced) autonomic arousal even under anesthesia [31,32,33], including an increase in blood norepinephrine (*i.e.*, a natural vasoconstrictor in the skin) level [31]. Thus, the increase in blood norepinephrine level may well explain the sustained reduction in skin temperature in both hands (a sign of systemic vasoconstriction) following topical application of mustard oil to one hand in humans [34]. Along this line, the overwhelming decrease in HbT in our rodent experiment may be attributed to the vasoconstriction in the scalp as a consequence of a systemic increase in blood norepinephrine level following noxious stimulation. Interestingly, the innocuous brushing also induced a decrease in regional HbT although to a much less extent compared to noxious stimulation. In light of the fact that the pattern of autonomic arousal varies with emotions [35,36], brushing may cause an autonomic arousal, despite being at a moderate level, as if in the awake state, rats might feel unpleasant or annoying when brushing was applied to the hindpaw.

Together with our results and a speculation on autonomic arousal, we argue that our setup is able to capture cerebrovascular activity during pain processing in rats, but a substantial scalp-effect cannot be ignored. As the hemodynamic changes during an early period (0–10 min) from a middle-posterior ROI in the intact-scalp group resembled the results from the no-scalp group [12] in both spatial and temporal aspects, these regional hemodynamic changes appeared to originate from the brain rather than from the scalp skin. Thus, it is suggested that our multi-channel system is able to assess a genuine cerebrovascular response during functional activation in rats with the scalp. However, it is important to note that the scalp appeared to have a substantial influence on fNIRI signal, indicated by the completely different responses between the no-scalp and intact-scalp groups during brushing, pinching, and the late period after formalin injection. Both the positive and negative results suggested that the validity of our fNIRI results relies on the strength of signal contrast between the cerebrovascular activity and vascular activity in the scalp skin.

## 5. Conclusions

Our results suggested that the scalp can profoundly interfere with fNIRI signal following noxious as well as innocuous stimulation. While using tomographic algorithm and multi-channel system can improve the spatial resolution of fNIRI, the effect of the scalp should still be considered as a critical factor in image reconstruction and data interpretation.
